# Identification of Pinosylvin in *Pinus nigra* subsp. *laricio*: A Naturally Occurring Stilbenoid Suppressing LPS-Induced Expression of Pro-Inflammatory Cytokines and Mediators and Inhibiting the JAK/STAT Signaling Pathway

**DOI:** 10.3390/ph16050718

**Published:** 2023-05-09

**Authors:** Maria Rosaria Perri, Michele Pellegrino, Mariangela Marrelli, Stefano Aquaro, Fabiola Cavaliere, Fedora Grande, Maria Antonietta Occhiuzzi, Carmine Lupia, Claudia-Crina Toma, Filomena Conforti, Giancarlo Statti

**Affiliations:** 1 Department of Pharmacy, Health and Nutritional Sciences, University of Calabria, 87036 Rende, Italy; mariarosaria.perri@unical.it (M.R.P.); michele.pellegrino@unical.it (M.P.); stefano.aquaro@unical.it (S.A.); fabiola.cavaliere@yahoo.it (F.C.); mariaantonietta.occhiuzzi@unical.it (M.A.O.); filomena.conforti@unical.it (F.C.); giancarlo.statti@unical.it (G.S.); 2 Mediterranean Ethnobotanical Conservatory, 88054 Sersale, Italy; studiolupiacarmine@libero.it; 3 National Ethnobotanical Conservatory, 85040 Castelluccio Superiore, Italy; 4 Pharmacognosy Department, Faculty of Pharmacy, Vasile Goldis Western University of Arad, 87 L. Rebreanu Str., 310045 Arad, Romania; claudiatoma2004@yahoo.com

**Keywords:** HPLC, IL-6, inflammation, molecular docking, *Pinus* extract, RAW 264.7, resveratrol, TNF-α

## Abstract

Stilbenoids, a group of phytoalexin polyphenols produced by plants as a defence mechanism in response to stress conditions, are known for their anti-inflammatory potential. Pinosylvin, a naturally occurring molecule traditionally found in pinus trees, was here identified in *Pinus nigra* subsp. *laricio* var. *calabrica* from Southern Italy through HPLC analysis. Both this molecule and its well-known analogue resveratrol, the most famous wine polyphenol, were compared for their in vitro potential anti-inflammatory activity. Pinosylvin significantly inhibited the release of pro-inflammatory cytokines (TNF-α and IL-6) and NO mediator in LPS-stimulated RAW 264.7 cells. Moreover, its ability to inhibit the JAK/STAT signaling pathway was assessed: Western blot analyses showed a downregulation of both phosphorylated JAK2 and STAT3 proteins. Finally, in order to verify whether this biological activity could be attributed to a direct interaction of pinosylvin with JAK2, a molecular docking study was performed, confirming the capability of pinosylvin to bind the active site of the protein.

## 1. Introduction

Stilbene common scaffold is characterised by two aromatic rings connected by an ethylene moiety. Although the *E* isomer represents the most common configuration, stilbenes exist in two diasteroisomeric forms: *E*-1,2-diphenylethylene (*trans*) and *Z*-1,2-diphenylethylene (*cis*) as monomeric, dimeric, trimeric, oligomeric, polymeric form or as glycosides. They are abundant in Gnetaceae, Pinaceae, Cyperaceae, Fabaceae, Dipterocarpaceae, and Vitaceae families. Stilbenes are commonly produced by plants as defence mechanisms when they are subjected to stress conditions such as UV irradiation, heat, and fungi or bacterial infections [1,2]. Stilbenoids contained in plants, a group of phytoalexin polyphenols, have been largely used in ancient folk medicine for the treatment of several diseases such as stomachache, hepatitis, arthritis, skin inflammation, and urinary tract and fungal infections. In addition to an extraordinary cardioprotective effect called the “French paradox”, connected to the low incidence of coronary disease in people consuming a saturated fat-rich diet, they expressed a large spectrum of biological effects concerning anticancer, anti-inflammatory, antimicrobial, antifungal, and neuroprotective activities; moreover, they result in being potentially useful in the treatment of obesity and diabetes [3]. Pecyna and colleagues recently reviewed the role of the -OH group in the stilbene structure and how it is able to modulate biological effects: it seems that the presence of an -OH group inhibits the nuclear factor kappa B (NF-kB) activation induced by tumour necrosis factor (TNF). Structure-activity studies showed that the increasing -OH group presence is correlated with better antioxidant and cytotoxic activities [1,4].

Starting from these encouraging considerations, new and different stilbene-based derivatives were designed and synthesised with the aim to improve the bioavailability and to modulate the potential anticancer activity among a wide range of cancer cell lines. In fact, structures with a stilbene backbone are considered promising agents in the cancer prevention field [5].

Pinosylvin and resveratrol are two stilbenes with a very similar structure, as they only differ in an -OH group. Pinosylvin (*trans*-3,5-dihydroxystilbene) is a naturally occurring stilbenoid mainly found in *Pinus* genus plants. This precious molecule seems to be present in the heartwood of conifer trees in a very low amount (about 1–40 mg/g of dry weight) [6]. Pinosylvin showed its anti-inflammatory activity by inhibiting the PGE_2_ production mediated by COX-2 in RAW 264.7 cells treated with LPS (1 µg/mL) with an IC_50_ value of 10.6 µM, almost half of the IC_50_ value detected for resveratrol in the same experiment (IC_50_ = 20.8 µM) [7]. According to Kimivaki and co-workers, pinosylvin is able to shift macrophages polarization from the pro-inflammatory M1 phenotype to the anti-inflammatory M2 phenotype, resulting in inflammation resolution and repair in J774 murine and U937 human macrophages stimulated with LPS [8].

Resveratrol (3,4′,5-trihydroxy-*trans*-stilbene) is a phytoalexin mainly found in grapes, red wine, peanuts, mulberries, bilberries, and cranberries. Commonly, resveratrol occurs in nature in its *trans* form, while *cis*-resveratrol is considered unstable and not commercially available. Only a few plant species are able to produce stilbenes as products, and the biosynthetic pathway follows the shikimate pathway: starting from phenylalanine, which is subjected to various enzymatic reactions, the intermediate *p*-coumaryl-CoA is produced, leading, in the presence of malonyl CoA and stilbene synthase, to the production of *trans*-resveratrol via aldol reaction [2,9].

Inflammation is a phenomenon triggered in response to pathogen attack and tissue injury. Immune cells (macrophages, leukocytes, neutrophils, and mast cells) release inflammatory mediators such as tumour necrosis factor (TNF-α) and interleukins (IL) that repair local damages and favour the expression of selectins and integrins [4]. Macrophages, in particular, are considered the key players in the immune system response in case of infection and inflammation: they can act as phagocytes, secrete cytokines, chemokines, pro-inflammatory factors, but also anti-inflammatory ones able to resolve the inflammation [8]. In summary, macrophages, by releasing cytokines and growth factors, play a leading role in the inflammatory process [10]. The Janus kinase/signal transducer and activator of transcription (JAK/STAT) signaling pathway is involved in many different biological processes strictly connected to cell proliferation, differentiation, apoptosis, and immune regulation. Currently, the research concerning this pathway focuses on anti-inflammatory activity and chemoprevention. Some ligands, mainly cytokines, bind to their receptor and activate JAK. JAK activation leads to tyrosine phosphorylation of the receptor, which in turn forms a STATs docking site. Phosphorylate JAKs are now able to phosphorylate STATs in the cytoplasm, which form homo- and heterodimers, translocate into the nucleus, and bind specific DNA sequences in order to activate or inhibit the transcription of a large variety of target genes and the expression of cytokine-responsive genes [11].

The JAK family includes four members: JAK1, JAK2, JAK3, and Tyk2 with over 1000 amino acids. The downstream target of JAKs is STAT. STAT comprises seven members with a molecular weight ranging from 79 to 113 kDa: STAT1, STAT2, STAT3, STAT4, STAT5A, STAT5B, and STAT6. More than 50 cytokines and growth factors, such as hormones, interferons, and interleukins, have a recognised role in the JAK/STAT signaling pathway modulation. It is possible to target the JAK/STAT cascade at three different levels by using cytokine or receptor antibodies, or JAK and STAT inhibitors [12,13].

Natural products and their derivatives are able to interfere with cancer initiation and progression and are able to target and downregulate several different signaling pathways whose activation results in abnormal cell proliferation. A group of phytochemicals such as taxanes, vinka alkaloids, and derivatives of podophyllotoxin, roscovitine, and camphotecine are widely used for cancer treatment, as many others are currently under study. Among these, phenolic compounds, including phenolic acids, lignans and stilbenes, are the most promising phytochemicals for the research and development of new anticancer drugs [14].

Curcumin, oleanolic acid, artemisinin, capsaicin, caffeic acid, berbamine, and epigallocatechin gallate showed interesting effects in affecting the above-mentioned pathway, but among others, resveratrol stands out for its chemopreventive and chemotherapeutic effects. In fact, resveratrol acts at a molecular level by targeting inflammatory cytokines and NF-kB; on the other hand, the immune response is regulated by resveratrol through the STAT1, STAT3, and NF-kB pathway suppression. Resveratrol inhibits the production of pro-inflammatory mediators and the modification of immune cells [15]. In human epidermoid carcinoma (A431) cells, resveratrol inhibits JAK phosphorylation and, consequently, it blocks STAT1 phosphorylation. In other specific cancer cells, it is able to inhibit the Src tyrosine kinase function and the STAT3 activation. The use of phytoconstituents, vitamins, and minerals is an open road for the so-called “cancer chemoprevention” [16,17,18]. Resveratrol exerts its anti-inflammatory activity by inhibiting the production of TNF-α and interleukin-1β (IL-1β) and through the induction of anti-inflammatory heme oxygenase-1 (HO-1) in RAW264.7 macrophages. Moreover, it is able to inhibit the transcription of interleukin-6 (IL-6), NF-kB, and the JAK/STAT signaling pathway. The inhibition of NF-kB determines low levels of IL-6 secretion; this effect results in a decreased STAT3 activation in macrophages [19].

Pinosylvin is traditionally identified in *Pinus nigra* and *Pinus sylvestris* sapwood and heartwood [20,21]. The commonly known black pine, *Pinus nigra* Arnold, belonging to the Pinaceae family, is a species widely distributed around the Mediterranean basin, from Europe to Asia, crossing through Crimea, Morocco, and Algeria. *Pinus nigra* species can be classified into six subspecies, including *Pinus nigra* subsp. *laricio* (Poiret) Maire, typical of the Southern Italy flora (Calabria and Sicily) and Corsica [22,23]. Two varieties of *Pinus nigra* subsp. *laricio*, in fact, are commonly known: the Corsican pine (var. *corsicana*) and the Calabrian pine (var. *calabrica*): the last one is endemic to Southern Italy, extending from Calabria to Sicily [24]. The tree is about 50 m high, with a dark bark and a linear trunk [25].

In this frame, starting from the already well-known anti-inflammatory potential of resveratrol, the aim of this work was to examine the biological features of pinosylvin, a still understudied resveratrol analogue. Here, its potential anti-inflammatory activity, in terms of pro-inflammatory cytokines and mediator inhibition, anti-inflammatory cytokine release, as well as the ability to inhibit the JAK/STAT signaling pathway, was deeply investigated and compared with that of resveratrol, in order to assess if similar structures could determine similar biological properties.

## 2. Results and Discussion

### 2.1. HPLC Analysis

**Figure 1:**
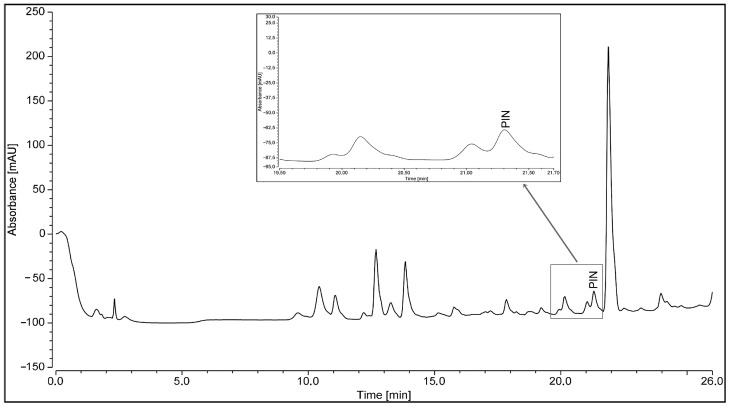
HPLC chromatogram of *Pinus nigra* subsp. *laricio* knotwood extract: pinosylvin (PIN) identification.

The stilbene pinosylvin was researched and identified in the knotwood hydrophilic extract of *Pinus nigra* subsp. *laricio* var. *calabrica* (PN2), as reported in the HPLC chromatogram (Figure 1). Pinosylvin was recognised by comparing retention time (RT) and UV-Vis spectra features with that of commercial pinosylvin (Sigma Aldrich s.p.A, Milan, Italy), spiked and used as an external standard (Figure S1).

Vek and colleagues described the presence of pinosylvin and pinosylvin monomethyl ether within the extracts of Scots pine or *Pinus sylvestris* and Black pine or *Pinus nigra* from Slovenia. Both samples showed comparable amounts of pinosylvin monomethyl ether (33.49 ± 11.83 and 31.53 ± 21.13 mg/g of dry weight in *Pinus sylvestris* and *Pinus nigra*, respectively) while significantly different concentrations of pinosylvin were assessed among the two investigated knotwoods (6.62 ± 1.60 and 4.26 ± 2.42 mg/g of dry weight for *Pinus sylvestris* and *Pinus nigra*, respectively) [23]. Ioannidis and colleagues investigated 260 *Pinus nigra* tree specimens belonging to the Peloponnese area (Greece). The major identified stilbenes were pinosylvin monomethyl ether, pinosylvin, and pinosylvin dimethyl ether [17]. According to Hovelstad and colleagues, pinosylvin was assessed in *Pinus sylvestris* from Norway in amounts ranging from 0.2–2/2–8% (*w*/*w*) [24]. Pinosylvin was also recovered in *Pinus sylvestris* knots from Slovenia and Finland obtained through accelerated solvent extractor [25].

### 2.2. Effects on LPS-Induced Release of Pro-Inflammatory, Anti-Inflammatory Cytokines and NO Mediator and Assessment of Cell Viability in RAW 264.7 Cells

**Figure 2:**
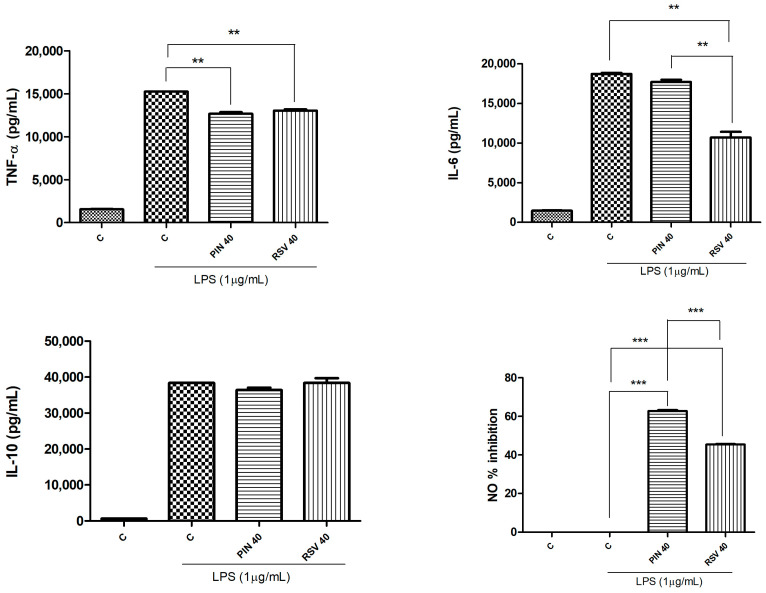
Decreased pro-inflammatory cytokines production (TNF-α and IL-6) and inhibition of NO mediator release by pinosylvin and resveratrol (used as positive control). Significant differences among investigated samples: ** *p* < 0.01; *** *p* < 0.001 (Student’s *t*-test). PIN 40, pinosylvin 40 µM; RSV 40, resveratrol 40 µM.

Macrophage cells, if properly activated and stimulated through endotoxin, lipopolysaccharides (LPS) and cytokines are able to trigger the inflammatory cascade by producing cytokines, chemokines, and through the activation of pro-inflammatory genes transduction [26]. In this case, commercial standards of pinosylvin and resveratrol were used for biological assay. Reference compounds, in fact, at the same final concentration (40 µM), were tested for their ability to inhibit the production of pro-inflammatory cytokines (TNF-α, IL-6) and NO mediator and to induce the release of the anti-inflammatory cytokine IL-10 in RAW264.7 cells stimulated with LPS, a Gram-negative bacteria cell wall constituent. As Figure 2 showed, both the molecules significantly inhibited the production of TNF-α if compared to control (*p* < 0.01, Student’s *t*-test).

**Figure 3:**
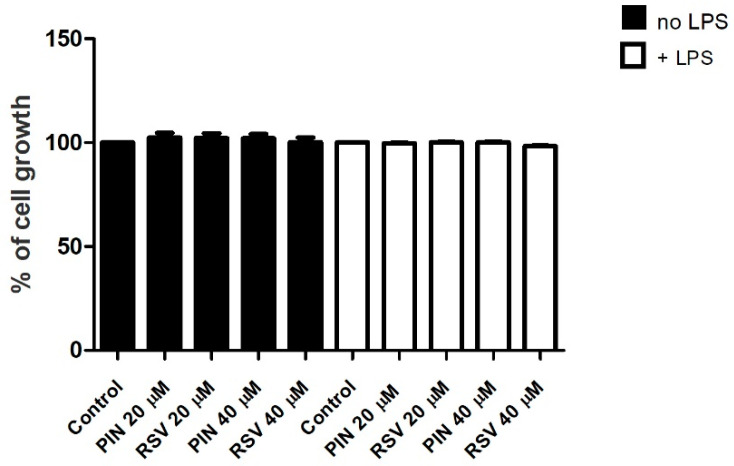
Results were expressed as mean ± S.D. (*n* = 4). Cell viability was showed as percentage of cell proliferation. PIN pinosylvin; RSV, resveratrol, LPS, lipopolysaccharide.

In regards to the inhibition of IL-6 production, resveratrol was statistically significant both versus control and pinosylvin (*p* < 0.01, Student’s *t*-test). On the contrary, pinosylvin exerted the best NO inhibition, with an inhibition percentage higher than 60%, statistically significant if compared to both control with LPS and resveratrol (*p* < 0.001, Student’s *t*-test, Figure 2). Then, in order to exclude the possibility that the inhibition activity was due to cell death, an SRB test was performed. As reported in Figure 3, none of the tested samples affected cell viability.

### 2.3. Effects on LPS-Activated JAK/STAT Signaling Pathway in RAW 264.7 Cells

**Figure 4:**
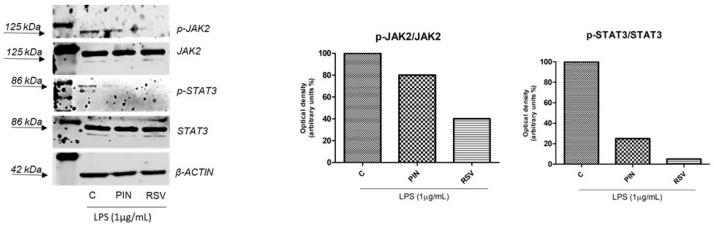
Downregulation of phosphorylated JAK2 and STAT3 proteins in RAW 264.7 cells previously stimulated with LPS (1 µg/mL) by pinosylvin 40 µM and resveratrol 40 µM (used as positive control). Treated and stimulated cells were lysed and equal amounts of total cellular extracts were run. β-Actin was used as loading control. Densitometric analyses of phosphorylated versus total proteins, previously normalised versus β-Actin were represented. The histograms refer to the reported densitometric analysis. PIN, pinosylvin; RSV, resveratrol, LPS, lipopolysaccharide.

The ability of pinosylvin to inhibit the phosphorylation of JAK2 and STAT3 proteins was assessed through Western blot analyses in RAW 264.7 macrophages. Cells were pretreated with samples and stimulated with LPS (1 µg/mL). As shown in Figure 4, pinosylvin downregulated both JAK2 e STAT3 phosphorylated proteins but, in both cases, resveratrol, used as reference compound, worked better, showing an excellent p-STAT3 inhibitory activity.

The potential anti-inflammatory activity of pinosylvin was further investigated over the last few years. Park and colleagues demonstrated that pinosylvin was able to modulate the production of prostaglandin E_2_ (PGE_2_) and NO via inhibition of COX2 and iNOS [27]. According to Erasalo and colleagues, pinosylvin inhibited the pro-inflammatory cytokine IL-6 with an IC_50_ value of 32.1 µM and monocyte chemotactic protein (MCP1) with an IC_50_ value equal to 38.7 µM. Moreover, in vivo studies showed how pinosylvin (30 mg/kg) was able to reduce paw edema induced by carrageenan in C57BL/6 mice model by downregulating the release of IL-6, MCP1 and NO if compared to the control group treated with phosphatidylinositol-3-kinase inhibitor LY294002, demonstrating the anti-inflammatory activity could be due to the PI3K/Akt pathway inhibition [28]. Park and colleagues showed that pinosylvin significantly inhibited NO production in RAW 264.7 cells with an IC_50_ value of 39.9 µM, a very interesting result if compared to the positive control (L-NAME, IC_50_ = 30.7 µM) and downregulated Interferon Regulatory Factor-3 (IRF-3) and Interferon-E (IFN-E). The same research group hypothesised that this activity could be due to JAK phosphorylation inhibitory activity [29].

Regarding resveratrol, its potential anti-inflammatory activity has been investigated over the years. According to Ma and colleagues, resveratrol, extracted and isolated from *Polygonum cuspidatum* species, was able to inhibit the release of IL-6 pro-inflammatory cytokine, downregulate STAT1 and STAT3 phosphorylation, and modulate NF-kB translocation into the nucleus in RAW 264.7 cells previously activated with LPS [30]. Moreover, Chung and colleagues demonstrated that the above-mentioned molecule inhibited the NO release caused by IFN-γ activity [31]. Furthermore, resveratrol seemed to be able to modulate the release of pro-inflammatory cytokines via downregulation of the JAK/STAT/RANKL signaling pathway [32].

### 2.4. Molecular Docking

JAK2 shares the same structural organization with other members of the JAK family, which essentially includes a C-terminal JH1 (JAK-1 homology) PTK domain next to a kinase-like domain (JH2), involved in regulating the PTK activity of JH1, and five additional JAK homology domains (JH3-JH7), among which the well-defined band F ezrin-radixin-moesin homology (FERM) domain (JH7) appears to be necessary for the interaction of JAKs with their related receptors and regulatory proteins [33,34]. As expected, the crystal structure of the human JAK2 PTK domain (residues 843–1132) also shows the typical protein kinase structural architecture, including a small and flexible N-terminal lobe (residues 840–931) comprising a five-stranded antiparallel β-sheet and an α-helix and a large C-terminal lobe (residues 932–1132), comprising eight α-helices, three 3/10 helices, and three pairs of antiparallel β-strands [35]. The N-lobe, which facilitates the activation and regulation of ATP/ADP binding and release, is connected by a hinge region to the C-lobe [36]. The JAK2 binding site, as reported in the literature, contains several essential residues which include Met929, Leu855, Val863, Ala880, Val911, Leu983, Gly935, Tyr931, Glu930, Leu932, Asp939, Ser936, Arg980, Gly993, Asp994, Asn981, Asn859, Lys882, Phe860, and Asp976. Out of these, Leu855, Val863, Ala880, and Val911 constitute a group of hydrophobic residues present in the N-terminal lobe of JAK2 while Leu983 and Gly935 are pivotal residues in the C-terminal lobe and Met929 and Tyr931 in the hinge region of JAK2 [37].

**Figure 5:**
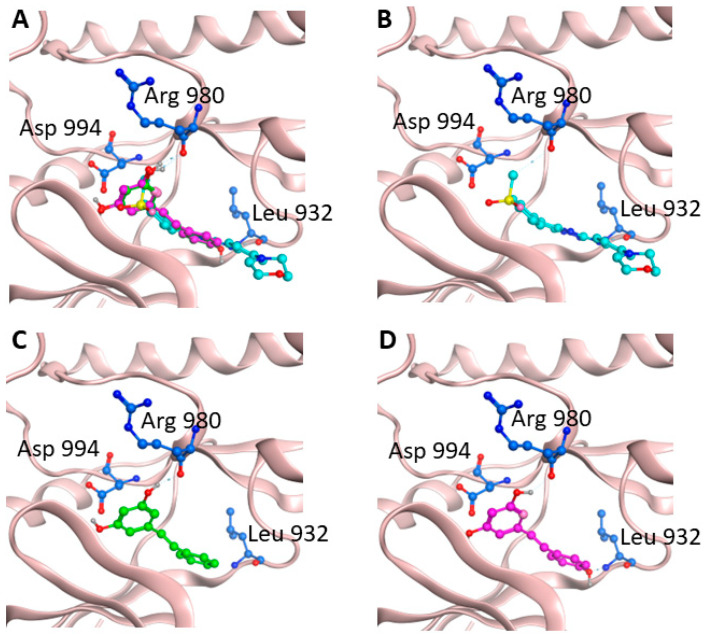
Ligand-binding pocket of the active site of JAK2. Protein backbone is represented in background as ribbons, and key protein residues are in blue. (**A**) Superimposed binding modes of triazolopyridine-based inhibitor (cyan); pinosylvin (green) and resveratrol (magenta). The ligands are also shown separately: (**B**) triazolopyridine-based inhibitor TP, (**C**) pinosylvin, and (**D**) resveratrol.

In this work, based on this knowledge and the encouraging results obtained in our biological experiments, we investigated the compatibility of the structure of pinosylvin with the active site of JAK2. To this aim, molecular docking studies have been assessed on a crystallographic structure of the protein catalytic portion retrieved from the Protein Data Bank (PDB code: 4AQC). In this structure, the protein is complexed with a triazolopyridine-based structure inhibitor (TP) [38]. As a first step, a re-docking experiment was carried out in order to calculate the binding energy value for the crystallographic ligand into the JAK2 binding site, obtaining a −10 kcal/mol value taken as a reference. Successively, pinosylvin was docked into JAK2, resulting in being able to fit into the protein binding site and interact with key residues. Resveratrol was also docked into the protein for comparison (Figure 5). As shown in Table 1, the estimated binding energy values were favourable for both compounds (−7.9 and −8.2 kcal/mol for pinosylvin and resveratrol, respectively), suggesting the stability of the complexes.

Pinosylvin was able to interact with the protein active site by establishing hydrogen bonds with Arg980 and Asp994 through its hydroxyl groups, and several hydrophobic interactions with other four key residues of the JAK2 active site. The same interactions were observed for resveratrol, which, however, thanks to an additional -OH group on its structure, was able to form a third hydrogen bond with Leu932. This further interaction is probably responsible for the slightly more favorable binding energy value recorded.

Overall, these findings indicate that pinosylvin could exert a direct effect on JAK2, confirming the evidence provided by Western blot analyses.

## 3. Materials and Methods

### 3.1. Chemicals

Dulbecco’s Modified Eagle’s Medium (DMEM), Fetal Bovine Serum (FBS), L-glutamine, penicillin/streptomycin, phosphate buffered saline (PBS), bovine serum albumin (BSA), protease inhibitors, trypan blue, lipopolysaccharide from *E. coli* (LPS), trichloroacetic acid (TCA), sulphorhodamine B (SRB), Tris base (Tris[hydroxymethyl]aminomethane), pinosylvin (HPLC, ≥97%), and resveratrol were purchased from Sigma-Aldrich S.p.a. (Milan, Italy). ECL System was from Bio-Rad. RAW 264.7 cells were purchased from ATCC, Glasgow, UK (No. TIB-71), employed Abs anti-phosphoJak2 (#PA538287), anti-Jak2 (#PA511267), anti-phosphoStat3 (#PA5121259), and anti-Stat3(#PA5120138) were from ThermoFisher Scientific. Anti-β-actin (AC-15; sc-69879) was from Santa Cruz Biotechnology, Inc., Heidelberg, Germany. Water and acetonitrile were HPLC-grade, while all other solvents were reagent-grade, obtained by VWR International s.r.l. (Milan, Italy).

### 3.2. Plant Material and Extraction Procedure

*Pinus nigra* subsp. *laricio* var. *calabrica* trees, fallen following a whirlwind in Sersale (Catanzaro) Southern Italy, were collected (leg., det. C. Lupia) and part of them were actually exhibited in the xylotheque (sample number 5) of the Mediterranean Ethnobotanical Conservatory of Sersale, Italy. *Pinus nigra* subsp. *laricio* knotwood, properly shredded and pulverised, was subjected to two different extraction processes through the Soxhlet apparatus: the first one in cyclohexane (110 °C, 6 h) (PN1: *Pinus nigra laricio* extract obtained through cyclohexane), and the second one in distilled water (110 °C, 6 h) (PN2: *Pinus nigra laricio* extract obtained through distilled water) [23].

### 3.3. HPLC Analysis

Pinosylvin was identified in *Pinus nigra* subsp. *laricio* extract (PN2) through a Vanquish high-performance liquid chromatography (HPLC) Instrument (ThermoFisher Scientific, Milan, Italy) associated with a VC-D11-A Diode Array Detector (DAD). Before the injection, standard and extract were properly filtered through a 0.45 µM membrane filter into a HPLC vials. The separation was carried out on an Acclaim^TM^ 120 C18 column 3 µm 120 Å (3.0 × 150 mm). The mobile phases were water (A) and acetonitrile (B), both containing 0.1% acetic acid with the following gradient: 0 min 95% B, 23 min 30% A and 70% B, 25 min 5% A and 95% B, 26 min 95% A and 5% B. The flow rate and the column temperature were 0.4 mL/min and room temperature, respectively. Pinosylvin, used as standard, was detected at 272 nm and the identification within the extract was made by comparing retention times and UV-Vis spectra features.

### 3.4. Cell Culture

RAW 264.7 cells were purchased by ATCC, Glasgow, UK (No. TIB-71) and cultured in Dulbecco’s Modified Eagle’s Medium (DMEM) implemented with 10% Fetal Bovine Serum, 1% Glutamine and 1% Penicillin/Streptomycin. Cells, incubated at 37 °C and 5% CO_2_ atmosphere, were tested monthly for mycoplasma (MycoAlert Mycoplasma Detection Kit, Lonza, Switzerland).

### 3.5. Cell Viability (SRB) Assay

In order to evaluate the cytotoxic effects, 3000 cells/well were seeded into a 96-well plate and incubated for 24 h in order to favour attachment. The day after, a treatment with pinosylvin and resveratrol 40 µM, followed by stimulation with LPS (1 µg/mL), was conducted and cells were incubated for a further 24 h. When time ran out, cells were treated with trichloroacetic acid (TCA) for 1 h at 4 °C and a sulphorhodamine B (SRB) solution was added for staining. At the end of the process, cells were washed three times with acetic acid solution 1% and air dried. Then, the dye was solubilised with 100 µL/well of 10 mM Tris base (Tris [hydroxymethyl]aminomethane). Absorbance was measured at 540 nm at a microplate reader (Stat fax 3200, Awareness Technology Inc., Palm City, FL, USA) [39].

### 3.6. Cytokine Measurements and Nitrite Analysis

For the experiments, 3 × 10^5^ cells/well were seeded into a 24-well plate and incubated. At 24 h later, cells were treated with pinosylvin and resveratrol 40 µM for 30 min, then stimulated with LPS (1 µg/mL) and incubated again. The day after, media was collected: TNF-α, IL-6 and IL-10 levels were evaluated through ELISA kits (TermoFisher Scientific, Bender MedSystem GmbH, Vienna, Austria) as indicated by manufacturer’s instruction, while the presence of nitrite was evaluated by mixing the surnatant with the same volume of Griess reagent. Absorbance was measured at 550 nm with a spectrophotometer (Stat fax 3200 Awareness Technology Inc., Palm City, FL, USA) [39].

### 3.7. Western Blot Analyses

RAW 264.7 cells treated and activated with LPS were lysed after 2 h in RIPA lysis buffer [20 mM Tris-HCl (pH 7.5), 150 mM NaCl, 1 mM Na2 EDTA, 1 mM EGTA, 1% NP-40, 1% sodium deoxycholate, 2.5 mM sodium pyrophosphate] implemented with protease inhibitor cocktail and phosphatase inhibitor. Equal amounts of protein lysed extracts were run on SDS-PAGE 11% gel and electroblotted onto a nitrocellulose membrane. Proteins were detected with polyclonal and monoclonal antibodies and recognised by IRDye secondary Abs (LI-COR Corporate, Milan, Italy). Western blot images were accomplished through Odyssey FC Imaging System (LI-COR Corporate) and showed with relative molecular weight markers; densitometry readings/intensity ratio were assessed through Image J software (Rasband, W.S. ImageJ, U.S. National Institutes of Health, Bethesda, MD, USA) [39].

### 3.8. Molecular Docking Studies

Molecular docking studies were performed on the crystallographic structure of the JAK2 catalytic portion in complex with triazolopyridine-based inhibitor ((TP, 8-(4-methylsulfonylphenyl)-*N*-(4-morpholin-4-ylphenyl)-[1,2,4]triazolo[1,5-*a*]pyridin-2-amine) corresponding to PDB entry 4AQC [38]. Resveratrol and pinosylvin molecular structures were built by using Avogadro [40] modeling software and docking calculations were carried out by using AutoDock Vina 1.1.2 [41]. Preliminary conversion of the structures from the PDB format was carried out by the graphical interface AutoDock Tools 1.5.6 [42]. During the conversion, polar hydrogens were added to the crystallographic enzyme structures, whereas apolar hydrogens of the ligands were merged to the carbon atom they are attached to. Full flexibility was guaranteed for the ligands, resulting in five active torsions for resveratrol and four for pinosylvin. The binding modes of the ligands were analysed through visual inspection and intermolecular interactions were evaluated by using the automated protein–ligand interaction profiler, PLIP [43] and MOE 20018.01 (Molecular Operating Environment) (Chemical Computing Group ULC, 1010 Sherbooke St. West, Suite #910, Montreal, QC, Canada, H3A 2R7 in, 2018).

### 3.9. Statistical Analysis

Experiments were performed in triplicate (*n* = 3), with the exception of cell viability assay, which was run in quadruplicate (*n* = 4). Data were expressed as mean ± S.D. Normality of data and homogeneity of variances were assessed through D’Agostino–Pearson’s K2 test and Levene’s test, respectively. Statistical differences were assessed by Student’s *t*-test (Graph-Pad Prism Software 5, San Diego, CA, USA). Western blot analyses were analysed through Image J (Rasband, W.S. ImageJ, U.S. National Institutes of Health, Bethesda, ML, USA).

## 4. Conclusions

Starting from the encouraging and well-determined anti-inflammatory activity of resveratrol, the aim of this work was to investigate the potential of one of its analogues, pinosylvin, a naturally occurring stilbenoid traditionally extracted from plants belonging to the *Pinus* genus. The stilbenoid was identified within the *Pinus nigra* subsp. *laricio* var. *calabrica* species, widely spread in the woods in Southern Italy. In this study, the activity of pinosylvin in inhibiting release and production of pro-inflammatory cytokines such as TNF-α and IL-6, NO mediator, and the phosphorylation of JAK2 and STAT3 proteins was deeply investigated in order to establish its potential anti-inflammatory activity. Moreover, as shown by molecular docking, it fits into the JAK2 protein active site with a favourable binding energy value (−7.9 kcal/mol). These findings were then validated through comparison with resveratrol. The presence of an additional hydroxyl group on the second benzene ring of resveratrol, besides justifying a more favourable binding energy, could suggest that the formation of a more stable ligand–protein complex is also responsible for its improved biological properties. Nonetheless, the evidence from our experiments suggests that pinosylvin exhibits a remarkable potential anti-inflammatory activity, confirming the hypothesis that chemical structure similarities allow for similar biological properties.

## Figures and Tables

**Table 1:** Binding energies for pinosylvin and resveratrol and key protein residues of JAK2 receptor interacting with the ligands.

Ligand	Structure	Binding Energy kcal/mol	INTERACTIONS				
			Hydrogen Bonds			Hydrophobic Bonds	
			Residues	Distance Å		Don-Angle	Residues
				H-A	D-A		
Pinosylvin	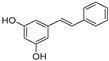	−7.9	Arg980	2.02	2.97	166.10	Leu855 Val863 Ala880 Leu983
			Asp994	2.77	3.47	128.82	
Resveratrol	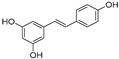	−8.2	Leu932	1.93	2.82	148.94	Leu855 Val863 Ala880 Leu983
			Arg980	2.26	3.02	134.20	
			Asp994	2.98	3.59	121.71	

## Data Availability

The data presented in this study are available in the article.

## Supplementary Materials

The following supporting information can be downloaded at https://www.mdpi.com/article/10.3390/ph16050718/s1. Figure S1: HPLC chromatogram of pinosylvin (PIN) standard.
