# Supplementary figures and images for: Identification of Pinosylvin in Pinus nigra subsp. laricio: A Naturally Occurring Stilbenoid Suppressing LPS-Induced Expression of Pro-Inflammatory Cytokines and Mediators and Inhibiting the JAK/STAT Signaling Pathway
Source: Pharmaceuticals (Basel). 2023 May 9;16(5):718. doi: 10.3390/ph16050718 (PMC10221723; doi:10.3390/ph16050718)

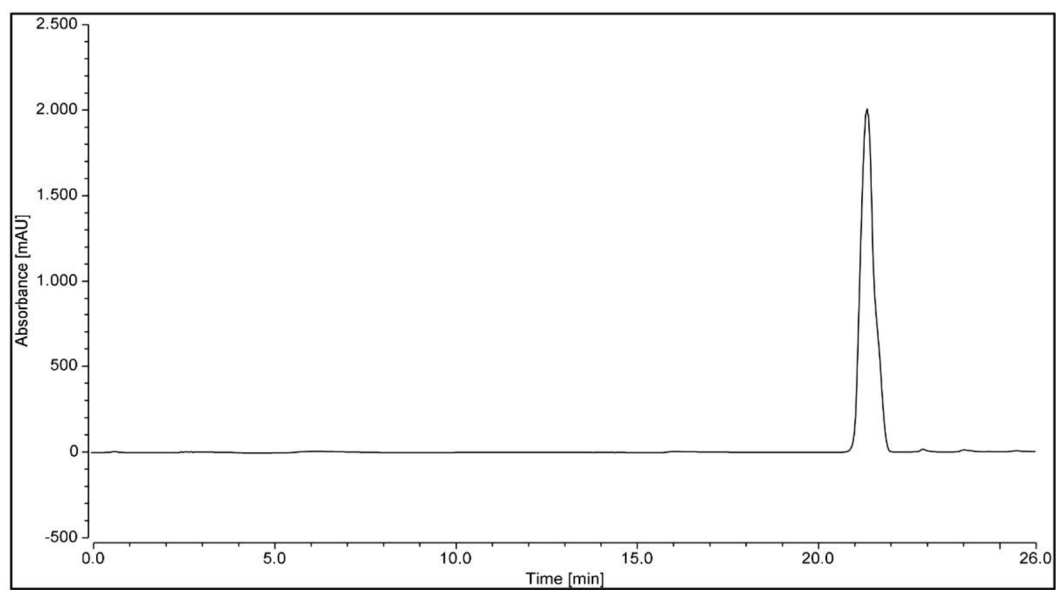

**Figure S1.** HPLC chromatogram of pinosylvin (PIN) standard.

Supplement: Supplementary file 1 [file pharmaceuticals-16-00718-s001.zip › pharmaceuticals-2349646-supplementary.pdf]
